# IgA pemphigus following COVID-19 vaccination: A case report

**DOI:** 10.1177/2050313X231181022

**Published:** 2023-06-14

**Authors:** Rafael Paolo Lansang, Esiahas Amdemichael, Dusan Sajic

**Affiliations:** 1 Michael G. Degroote School of Medicine, McMaster University, Hamilton, ON, Canada; 2 Faculty of Medicine, McMaster University, Hamilton, ON, Canada

**Keywords:** Pemphigus, IgA pemphigus, COVID-19, COVID-19 vaccination, adverse effects, Moderna vaccine

## Abstract

A patient presented with pruritic lesions on their back and leg after COVID-19 vaccination. Biopsy and direct immunofluorescence were consistent with IgA pemphigus—likely caused by the COVID-19 vaccine.

## Introduction

Messenger RNA (mRNA) COVID-19 vaccines have been shown to cause a variety of secondary cutaneous reactions in a small percentage of patients. Unspecific injection-site reactions are most common, particularly localized erythema.^1^ Other somewhat commonly reported reactions include urticarial eruptions, pityriasis rosea, and herpes zoster.^1,2^ To date, 36 cases of autoimmune bullous diseases triggered by COVID-19 vaccines have been reported.^3^

## Case report

**Figure 1.**
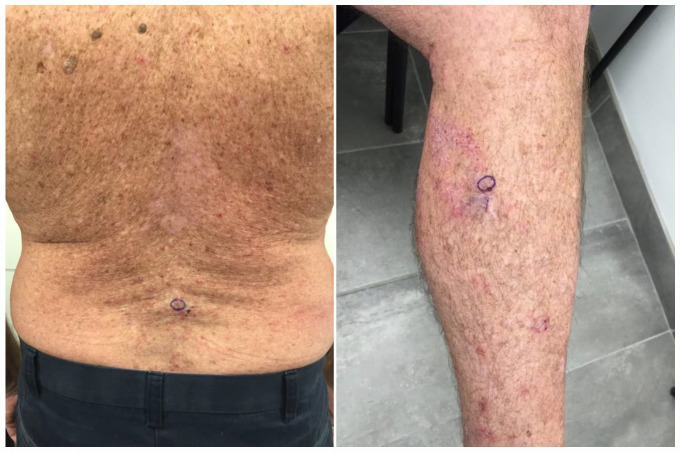
Biopsy-proven IgA pemphigus on patient’s back and left leg.

We report a 64-year-old male with a history of actinic keratosis and squamous cell carcinoma who came to the clinic complaining of extremely itchy spots and a diffuse rash along their back and left leg approximately 20 days after receiving a dose of the Moderna COVID-19 vaccine. The patient had also received a dose of the AstraZeneca COVID-19 vaccine approximately 3 months prior. Punch biopsy showed a spongiotic inflammatory process with eosinophils on the left lower leg and acantholytic dermatosis on the back (Figure 1). Direct immunofluorescence showed epidermal intercellular fluorescence for IgG, IgA, and C3 in keeping with IgA pemphigus. Given the temporal association and the lack of new medications or supplements started by the patient, it was determined that the COVID-19 vaccine was likely to be the cause. The patient was prescribed supportive care including compounded topical 0.25% menthol and 0.25% camphor in 0.1% betamethasone valerate and oral rupatadine to manage his pruritus. Further treatment with intramuscular triamcinolone was initiated but was discontinued due to improvement in the condition and concerns regarding side effects. The patient is continuing with topical treatments which they feel are effective enough for symptom management. The rash has improved and is progressing well. The patient provided informed consent to publish this case and the associated images.

## Discussion

A growing number of reactions to COVID-19 vaccinations—both systemic and cutaneous—have been reported. To date and to our knowledge, 36 autoimmune bullous conditions induced by COVID-19 infection have been previously reported—including bullous pemphigoid, linear IgA bullous dermatosis, pemphigus vulgaris, pemphigus vegetans, and pemphigus foliaceus.^3^ To our knowledge, there have been no previously published reports of IgA pemphigus following administration of a COVID-19 vaccine.

The etiology of IgA pemphigus is poorly understood, though it has been reported concomitantly with inflammatory conditions and internal malignancies.^4^ Typically, IgA pemphigus should be considered in patients presenting with a vesiculopustular eruption and/or circinate plaques with a predominantly truncal and extremity involvement, though definitive diagnosis is made with histopathology.^4^ Given the rarity of the disease, data on the clinical course and outcome of patients are limited. The majority of reported patients presented with a combination of vesicles, pustules, and circinate plaques, though a very small minority presented with only urticarial plaques, scars, or milia. The trunk and extremities were the most common areas of presentation, though a small minority presented with intertriginous, cephalic, generalized, or mucosal involvement.^4,5^ Management of IgA pemphigus can be quite recalcitrant and can often require systemic medications, such as oral dapsone, corticosteroids, vitamin A derivatives, and other disease-modifying antirheumatic drugs (DMARDs), such as cyclophosphamide, mycophenolate mofetil, and azathioprine. Scattered reports have also described management of refractory cases with infliximab, adalimumab, intravenous immunoglobulin (IVIG), and plasmapheresis.^4,5^ Given that our patient responded well to relatively conservative therapy, it can be suggested that vaccine-induced IgA pemphigus may present a more indolent course.

In summary, we want to present a case of biopsy-proven IgA pemphigus that developed in a patient after receiving a COVID-19 vaccine. Physicians should be aware about a growing number of adverse reactions that may occur following COVID-19 vaccination in susceptible patients. Given that IgA pemphigus is rare and may require aggressive immunosuppressive therapy in select patients, it is reassuring that this vaccine-induced case responded well to conservative management. The mild nature of our patient’s reaction to the vaccine shows that the risk–benefit equation of immunization toward future variants of COVID-19 should be considered together with patients. Preventive measures, such as prophylactic antihistamines prior to vaccination, may warrant further investigation in future research. Patients should also be advised to report any post-vaccination cutaneous developments as early as possible in order to initiate the most conservative treatment.
